# Intubation of non-difficult airways using video laryngoscope versus direct laryngoscope: a randomized, parallel-group study

**DOI:** 10.1186/s12871-019-0737-3

**Published:** 2019-05-15

**Authors:** De-Xing Liu, Ying Ye, Yu-Hang Zhu, Jing Li, Hong-Ying He, Liang Dong, Zhao-Qiong Zhu

**Affiliations:** Department of Anesthesiology, Affiliated Hospital of Zunyi Medical College, No. 149 Dalian Road, Zunyi, 563000 China

**Keywords:** Intubation, Anesthesia, Glottic exposure, Abdominal surgery

## Abstract

**Background:**

The video laryngoscope is recommended for intubating difficult airways. The present study aimed to determine whether the video laryngoscope can further improve intubation success rates compared with the direct laryngoscope in patients with non-difficult airways.

**Methods:**

In total, 360 patients scheduled for elective abdominal surgeries were randomly assigned to undergo intubation using either a video laryngoscope (*n* = 179) or a direct laryngoscope (*n* = 181). The following parameters were measured: mouth opening; thyromental distance; sternomental distance; shape angle of the tracheal catheter; and glottic exposure grade.

**Results:**

The percentage of patients with level I-II of total glottic exposure in the video laryngoscope group was 100% versus 63.5% in the direct laryngoscope group (*P* < 0.001). The one-attempt success rate of intubation was 96.1% using a video laryngoscope versus 90.1% using a direct laryngoscope (*P* = 0.024). The intubation success rate using a video laryngoscope was 100% versus 94.5% using a direct laryngoscope (*P* = 0.004). Immediate oropharyngeal injury occurred in 5.1% of patients intubated using a direct laryngoscope versus 1.1% using a video laryngoscope (*P* = 0.033). On postoperative day 1, obvious hoarseness was exhibited by 7.9% of patients intubated using a direct laryngoscope versus 2.8% using a video laryngoscope (*P* = 0.035). The grade of glottic exposure and catheter shape angle were independent risk factors for tracheal intubation failure. Thyromental distance, shape angle, glottic exposure time, and surgical position were independent risk factors for postoperative complications. Thyromental distance and glottic exposure time were independent risk factors for complications lasting > 2 days.

**Conclusions:**

Intubation using a video laryngoscope yielded significantly higher intubation success rates and significantly fewer postoperative complications than direct laryngoscopy in patients with non-difficult airways.

**Trial registration:**

Chinese Clinical Trial Registry. No: ChiCTR-IOR-16009023. Prospective registration.

## Background

The success rate of tracheal intubation in cases of difficult airways has increased significantly with the use of video laryngoscopes and improvements in the degree of glottic exposure [1–3]. The 2013 Guidelines from the American Association of Anesthesiologists for Difficult Airway Treatment recommended the video laryngoscope as the first choice after the failure of direct laryngoscope intubation [4]. Due to the unique lens design of most video laryngoscopes, in which the distal end is tilted upward and the angle with the horizontal plane is significantly larger than that of the direct laryngoscope, neck extension is not necessary after mastering the intubation skills. Moreover, during intubation, throat structure is always within the operator’s field of view, and cricoid pressure and external laryngopharyngeal operations are also diminished to reduce the incidence of throat injury [5, 6].

The video laryngoscope can reduce the complications of tracheal intubation associated with difficult airways; therefore, an analysis of its potential for completely replacing direct laryngoscopy is warranted. The present study aimed to compare the video laryngoscope with the direct laryngoscope for tracheal intubation in patients with non-difficult airways scheduled for elective abdominal surgeries.

## Methods

### Patients

The present study was approved by the Ethics Committee of the Affiliated Hospital of Zunyi Medical College (No. 2015062901). All patients were recruited from the Affiliated Hospital of Zunyi Medical College (Guizhou province, China) between April and December 2017. Before study enrollment, all participants provided informed written consent for participation. The trial was retrospectively registered at the China Clinical Trial Registration Center (www.chictr.org.cn, ChiCTR-IOR-16009023; Principal investigator, Liu Dexing; date of registration, August 14, 2016) before patient enrollment, and is not ongoing. Our study adheres to the CONSORT guidelines.

**Fig. 1:**
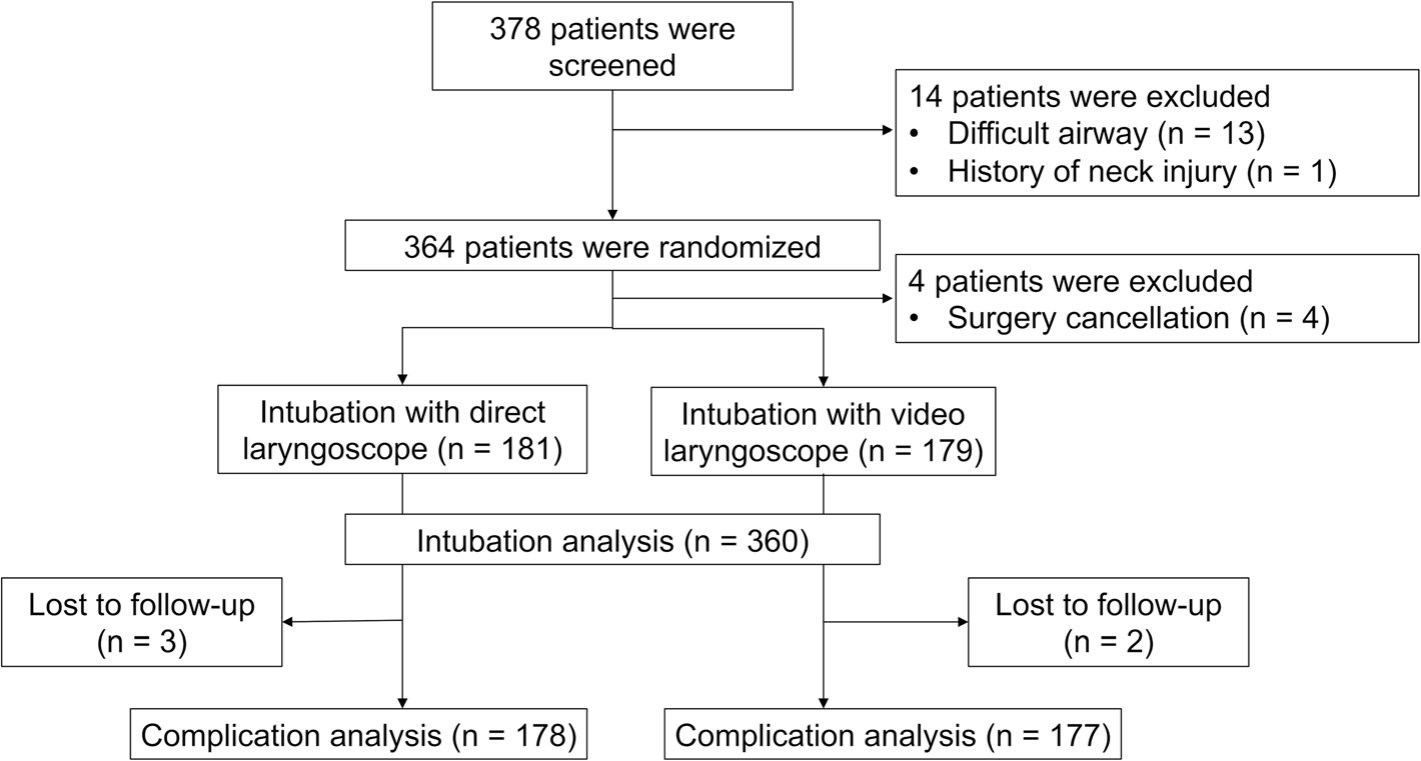
A flow chart illustrating patient inclusion

The inclusion criteria were: [1] age ≥ 18 years; [2] scheduled for elective abdominal surgery and; [3] required tracheal intubation under general anesthesia. Patients with a history of a neck injury, those with a difficult airway, those who had participated in other clinical trials within the previous 3 months or who had other contraindications to intubation, were excluded. Patients with any of the following conditions were predicted to have a difficult airway: [1] thyromental distance < 6 cm; [2] mouth opening < 3 cm; [3] cervical ankylosis; [4] a class IV Mallampati score. The patient inclusion process is illustrated in Fig. 1.

### Anesthesia protocol

After the patient entered the operating room, the thyromental distance and sternomental distance were recorded. Intravenous access was established and monitors were connected. Patients were monitored for electrocardiogram, non-invasive blood pressure, oxygen saturation, heart rate, and end-tidal carbon dioxide (PEtCO_2_). A reinforced endotracheal tube with an inner diameter of 7.0 mm was used for females, while a reinforced endotracheal tube with an inner diameter of 7.5 mm was used for males. Midazolam 2 mg, sufentanil 0.4 μg/kg, etomidate 0.3 mg/kg, and rocuronium 0.8 mg/kg were administrated intravenously. Tracheal intubation was performed 90 s after rocuronium injection. Mechanical ventilation was initiated after completion of intubation. The parameter settings included a tidal volume of 10 ml/kg and a respiratory rate of 12 breaths/min; respiratory parameters were adjusted according to PEtCO_2_ during the operation. Propofol and remifentanil were injected by the intravenous compound pump, with the use of sevoflurane to maintain anesthesia.

For intubation using a direct laryngoscope, the mirror handle was held in the left hand while the right hand was used to open the mouth. With the neck in the extended position, the laryngoscope lens was placed at the root of the epiglottis to expose the glottis. The exposure classification (Wilson-CL classification) was recorded. The tracheal tube, of which the shape angle was recorded, was then placed. If the number of consecutive intubation failures exceeded 2, a change to an alternative intubation method was performed or was attempted by another qualified intubation personnel. Glottic exposure grade, glottic exposure time, and total time of tracheal intubation were recorded.

**Fig. 2:**
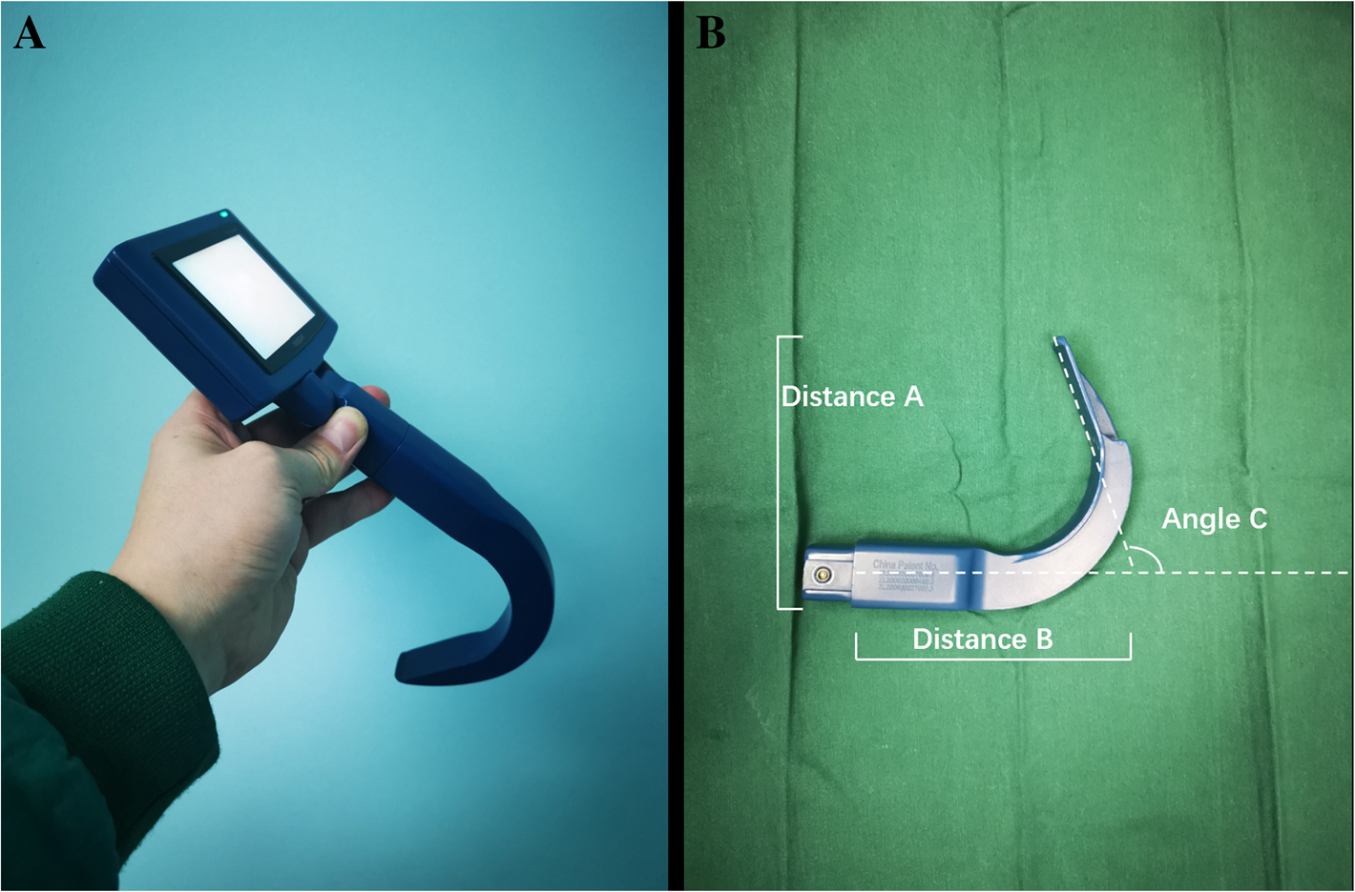
A picture of the video laryngoscope. **a** The video laryngoscope. **b** The parameters. Distance A, 11.5 cm. Distance B, 11.5 cm. Angle C, 120°

For intubation using a video laryngoscope (Fig. 2; TOSIGHT, Shanghai Jingshen Electronic Technology, China), the patient’s neck was not extended, and the remaining maneuvers were the same as those used for the direct laryngoscope.

In the direct laryngoscope group, a video laryngoscope was used upon two consecutive intubation failures with the direct laryngoscope. If the video laryngoscope also failed, intubation with an additional fiber bronchoscope is considered. In the video laryngoscope group, an additional fiber bronchoscope is used upon two consecutive intubation failures.

Before induction, the endotracheal tube was modified to be linearly shaped 1 cm after the cuff. The shape was adjusted by the operators according to their experience and the researchers did not interfere during such adjustments. The angle was traced on sterile non-woven fabric and measured.

### Patient evaluation

The basic information included: age; sex; weight; height, risk factors for difficult mask ventilation; American Society of Anesthesiologists (ASA) score, modified Mallampati grade; type of surgery; surgical position; and duration of surgical anesthesia. The primary outcomes included intubation success rate, intubation complications, intubation time, immediate intubation injury, and postoperative 7-days complications after, of which the definitions are presented in Table 1. The secondary outcomes included: anatomical parameters (mouth opening, thyromental distance, and sternomental distance); shape angle of the tracheal catheter; and glottic exposure according to the Wilson-C-L grading.

### Statistical analyses

The sample size calculation was based on the results of a pre-experiment. This pre-experiment performed to include the maximum sample size comprised the following tests: a superiority test of the success rate of intubation in one of the two groups, the non-inferiority test of glottic exposure time in both groups, and the non-inferiority test of the total intubation time in the two groups. Finally, a superiority test for the success rate of intubation in one of the two groups was performed as the calculation basis for sample size. It was assumed that the test delta (Δ) was 0.05, the loss to follow-up rate and drop-out rate was 20%, and the statistical power was 95%, with a significance level of α = 0.05. A total of 364 subjects were required; 182 subjects in each group participated in randomization. According to the work experience of the intubating anesthetist, the patients in each group were again randomly allocated into groups with a senior anesthetist or a junior anesthetist, with 91 cases in each group. More specifically, they were randomly divided into the following groups: video laryngoscope by a senior anesthetist; video laryngoscope by a junior anesthetist; direct laryngoscope by a senior anesthetist; and direct laryngoscope by a junior anesthetist.

Randomized comparisons were performed in this study. Before the patients were formally included in the trial, random numbers were generated by an individual other than the operator, and an opaque random envelope was prepared. After the patients were formally included and the statistics of their baseline data collated, the researchers, who did not participate in the operation, randomly grouped the patients according to the random number within the envelope. After the completion of surgery, the researchers submitted the case questionnaire with only the random numbers but hid the random groups from the follow-up staff who performed the follow-up investigation.

Continuous data are presented as mean and standard deviation and were compared using the Student’s *t*-test. Categorical data are presented as frequencies or percentages and were compared using the Chi-squared test. Logistic regression analysis was used to analyze risk factors for tracheal intubation failure and postoperative complications. All statistical analyses were performed using the SPSS version 21.0 (IBM Corporation, Armonk, NY, USA); *p* < 0.05 was statistically significant.

## Results

Among the 364 patients, 4 were excluded due to surgery cancellations. Therefore, a total of 360 patients were included in the study: 181 in the direct laryngoscope group and 179 in the video laryngoscope group. Of these patients, 290 were female and 70 were male. There were no statistical differences in patient sex, age, height, weight, body mass index, ASA grade, anatomical parameters such as mouth opening, and surgical information between the direct laryngoscope and video laryngoscope groups (*p* > 0.05) (Table 2).

The tracheal intubation operators included the following individuals. “Dr. A” was a senior anesthetist assigned to the video laryngoscope group and had 12 years’ clinical work experience. He had cumulatively completed or guided complete tracheal intubation in > 6000 cases and had provided video laryngoscope intubation training in > 30 cases before patients were included. “Dr. B” was a junior anesthetist assigned to the video laryngoscope group and had 5 years’ clinical work experience. He had cumulatively completed or guided complete tracheal intubation in > 1500 cases and had provided video laryngoscope intubation training in > 30 cases before patients were included “Dr. C” was a senior anesthetist assigned to the direct laryngoscope group and had 11 years’ clinical work experience. He had cumulatively completed or guided complete tracheal intubation in > 5000 cases. Finally, “Dr. D” was a junior anesthetist assigned to the direct laryngoscope group and had 5 years’ clinical work experience. He had cumulatively completed or guided complete tracheal intubation in > 1800 cases.

The percentage of patients with levels I-II of total glottic exposure in the video laryngoscope group was 100%, which was higher than that in the direct laryngoscope group (63.5%). Glottic exposure times in the video laryngoscope group were all shorter than those in the direct laryngoscope group. The total intubation time of the video laryngoscope group was also shorter than that of the direct laryngoscope group. In the direct laryngoscope group, the total intubation time of the senior anesthetists was shorter than that of the junior anesthetists (Table 3).

**Table 3:** Comparison of glottic exposure grading, glottic exposure time, and total intubation time in each group (%, s)

	n	Glottic exposure		Intubation	
		Glottic exposure [1, 2]	Glottic exposure (≥3)	Glottic exposure time (s)	Total intubation time (s)
Direct laryngoscope	181	115 (63.5)	66 (36.5)	15.7 (10.3,22.6)	49.9 (40.6,64.0)
Junior	91	57 (62.6)	34 (37.4)	16.4 (11.1,22.7)	55.2 (44.6,71.6)
Senior	90	58 (64.4)	32 (35.6)	14.3 (10.0,21.9)	44.8 (37.7,58.2)^d^
Video laryngoscope	179	179 (100)^a^	0^*a*^	9.5 (7.7,12.0)^a^	46.6 (41.6,53.5)^a^
Junior	91	91 (100)^b^	0^*b*^	9.1 (7.8,13.1)^b^	45.5 (40.1,53.2)^b^
Senior	88	88 (100)^c^	0^*c*^	9.7 (7.7,11.1)^c^	47.5 (42.5,53.9)

a: compared with group direct laryngoscopy, *P* < 0.05

b: compared with group direct laryngoscopy (junior), *P* < 0.05

c: compared with group direct laryngoscopy (senior), *P* < 0.05

d: compared with group direct laryngoscopy (junior), *P* < 0.05

**Table 1:** Main observational indicators and their definitions

Main observational indicator	Definition
Immediate intubation injury	Immediate mouth, pharynx and larynx injury, or incisor injury after intubation
Postoperative intubation complications	Postoperative intubation complications in patients including neck pain, pharyngeal pain, dysphagia, hoarseness, dysphonia, and dislocation of the cricoarytenoid joint.
Grade of voice hoarseness	
Mild	Conscious hoarseness
Moderate	Obvious hoarseness can be heard
Severe	Dysphonia
Glottic exposure time	Time between the cessation of oxygen supply until the glottis is exposed, and determines when it is possible to intubate
Total duration of tracheal intubation	Time between the cessation of oxygen supply until the waveform is confirmed with end tidal carbon dioxide monitoring

**Table 2:** Patient characteristics

	Direct laryngoscope (n = 181)	Video laryngoscope (n = 179)	*P*
Patient characteristic			
Male/female	31 (17.1)/150 (82.9)	39 (21.8)/140 (78.2)	0.264
Age (y)	41.7 ± 10.2	40.7 ± 10.9	0.313
Height (cm)	159.7 ± 6.6	159.8 ± 7.5	0.731
Weight (kg)	61.0 ± 10.6	59.7 ± 10.7	0.309
Body mass index (kg/m^2^)	23.9 ± 3.5	23.3 ± 3.3	0.158
ASA classification			
I	87 (48.1)	87 (48.6)	0.919
II	94 (51.9)	92 (51.4)	0.919
DMV risk factors	100 (55.2)/81 (44.8)	94 (52.5)/85 (47.5)	0.603
Mallampati			
1	40 (22.1)	49 (27.4)	0.246
2	128 (70.7)	117 (65.4)	0.276
3	13 (7.2)	13 (7.3)	0.977
Anatomical parameters			
Mouth opening (cm)	4.0 ± 0.6	4.0 ± 0.5	0.707
Thyromental distance (cm)	8.6 ± 1.3	8.5 ± 1.1	0.342
Sternomental distance (cm)	15.1 ± 2.0	15.0 ± 1.9	0.717
Type of surgery			
Gynecology	76 (42.0)	72 (40.2)	0.734
Hepatobiliary	100 (55.2)	100 (55.9)	0.906
Urology	2 (1.1)	1 (0.6)	1
Gastrointestinal	3 (1.7)	6 (3.4)	0.489
Surgical position			
Low head and high foot	71 (39.2)	66 (36.9)	0.645
High head and low foot	101 (55.8)	102 (57.0)	0.821
Supine position	9 (5.0)	10 (5.6)	0.794
Side position	0	1 (0.6)	0.497
Surgical site			
Upper abdomen	100 (55.2)	100 (55.9)	0.906
Midsection	2 (1.1)	1 (0.6)	1
Lower abdomen	79 (43.6)	78 (43.6)	0.989
Operation time (min)	80.5 ± 53.3	88.4 ± 73.1	0.724

**Table 4:** Comparison of tracheal intubation in each group (n, %)

	n	1 attempt	2 attempts	Overall success rate	Intubation failure within two times	
					Change anesthetist	Change equipment
Direct laryngoscope	181	163 (90.1)	8 (4.4)	171 (94.5)	3 (1.7)	7 (3.9)
Junior	91	82 (90.1)	4 (4.4)	86 (94.5)	2 (2.2)	3 (3.3)
Senior	90	81 (90.0)	4 (4.4)	85 (94.4)	1 (1.1)	4 (4.4)
Video laryngoscope	179	172 (96.1)^*a*^	7 (3.9)	179 (100)^*a*^	0	0^*a*^
Junior	91	86 (94.5)	5 (5.5)	91 (100)	0	0
Senior	88	86 (97.7)^*c*^	2 (2.3)	88 (100)	0	0

a: compared with group direct laryngoscopy, *P* < 0.05

c: compared with group direct laryngoscopy (senior), *P* < 0.05

**Table 5:** Comparison of complications on the first day after operation in each group (n, %)

	n	Oropharyngeal injury	Neck pain	Pharyngeal pain	Dysphagia	Hoarseness			Dysphonia	Dislocation of the cricoarytenoid joint
						Mild	Moderate	Severe		
Direct laryngoscope	178	9 (5.1)	5 (2.8)	35 (19.7)	2 (1.1)	44 (24.7)	14 (7.9)	0	2 (1.1)	0
Junior	91	5 (5.5)	1 (1.1)	20 (22.0)	1 (1.1)	23 (25.3)	10 (11.0)	0	0	0
Senior	87	4 (4.4)	4 (4.6)	15 (17.2)	1 (1.1)	21 (24.1)	4 (4.6)	0	2 (2.3)	0
Video laryngoscope	177	2 (1.1)^*a*^	2 (1.1)	24 (13.6)	1 (0.6)	37 (20.9)	5 (2.8)^*a*^	1 (0.6)	0	0
Junior	91	2 (2.2)	0	7 (7.7)^*be*^	0	21 (23.1)	2 (2.2)^*b*^	1 (1.1)	0	0
Senior	86	0	2 (2.3)	17 (19.8)	1 (1.2)	16 (18.6)	3 (3.5)	0	0	0

a: compared with group direct laryngoscopy, *P* < 0.05

b: compared with group direct laryngoscopy (junior), *P* < 0.05

e: compared with group glidescope (junior), *P* < 0.05

**Table 6:** Risk factors for ≥2 intubation failures, and postoperative intubation complications and complications lasting > 2 days

	Intubation failure within two times			Postoperative intubation complications			Complications lasting longer than two days		
	Univariate	Multivariate	OR (95% CI)	Univariate	Multivariate	OR (95% CI)	Univariate	Multivariate	OR (95% CI)
	P	P		P	P		P	P	
Types of laryngoscope	0.004	0.995	/				0.018	0.651	/
Glottic exposure grading	< 0.001	0.042	4.38 (1.06 to 18.1)				0.017	0.904	/
Angle of tracheal catheter	0.029	0.011	6.28 (1.53 to 25.84)	< 0.001	0.004	2.72 (1.38 to 5.38)			
Glottic exposure time				0.013	0.007	2.00 (1.21 to 3.31)	< 0.001	0.043	3.69 (1.04 to 13.11)
Thyromental distance				< 0.001	0.001	2.36 (1.40 to 3.99)	0.009	0.017	2.983 (1.22 to 7.32)
Surgical position				0.002	0.015	1.92 (1.14 to 3.24)			
Anesthesia time				0.006	0.587	/			
The time with catheter				0.001	0.209	/			
Total intubation time							0.002	0.451	/

In total, 172 (96.1%) patients in the video laryngoscope group were successfully intubated in one attempt, which was higher than that in the direct laryngoscope group (163 cases [90.1%]). The success rate in the senior group (97.7%) was higher than that in the junior group (94.5%). In the comparison of overall intubation success rate, all 179 patients (100%) in the video laryngoscope group were successfully intubated, which was higher than the 171 patients (94.5%) in the direct laryngoscope group. In the analysis of intubation failure, 7 (3.9%) patients in the direct laryngoscope group were switched to the video laryngoscope; however, there was no tool replacement in the video laryngoscope group; the difference between the two groups was statistically significant (Table 4).

Overall, 5 cases were lost to follow-up after the operation, and a total of 355 cases were included in the complication analysis: 5.1% of patients (9 cases) in the direct laryngoscope group experienced immediate oropharyngeal injury after intubation, which was higher than the 1.1% of patients (2 cases) in observed in the video laryngoscope group (*p* = 0.033). After intubation using the video laryngoscope, the incidence of postoperative pharyngeal pain in the junior anesthetist group was 7.7% (7 cases). This was lower than the 19.8% (17 cases) of cases assigned to the senior anesthetists in the same group and was also lower than the 22% (20 cases) of cases assigned to the junior anesthetists in the direct laryngoscope group. When comparing hoarseness on the first day after the surgery, 7.9% of patients (*n* = 14) in the direct laryngoscope group exhibited obvious hoarseness. This was higher than the 2.8% of patients (5 cases) seen in the video laryngoscope group. Ten cases (11.0%) with hoarseness were observed in the junior anesthetist group using the direct laryngoscope and this was higher than the 2 cases (2.2%) observed in the junior group using the video laryngoscope. There was no statistical difference between the two groups with regards to severe hoarseness or dislocation of the cricoarytenoid joint (Table 5).

Logistic regression was used to analyze independent risk factors for tracheal intubation failure. Analysis revealed that the grade of glottic exposure and catheter shape angle were independent risk factors for the failure of tracheal intubation; however, laryngoscopy classification was not an independent risk factor for intubation failure. Logistic regression revealed that thyromental distance, shape angle, glottic exposure time, and surgical position were the independent risk factors for postoperative complications, although laryngoscope classification was not an independent risk factor. Logistic regression demonstrated that thyromental distance and glottic exposure time were independent risk factors for tracheal intubation complications lasting > 2 days. Laryngoscopy classification was not an independent risk factor (Table 6).

## Discussion

The results of the present study demonstrated no significant differences between the senior and junior anesthetists in either group. This indicated that the glottic exposure rate was the same for the same intubation devices and it was not affected by the level of experience. The amount of muscle relaxant and onset time is directly related to glottic exposure during tracheal intubation. The induction and administration of muscle relaxants in this study and the wait time after drug administration were consistent with the pharmacokinetics and previous studies in both groups (rocuronium 0.8 mg/kg; tracheal intubation performed after 90 s of rocuronium injection) [7, 8]. However, the percentage of patients with Wilson-Cormack-Lehane classification levels I-II of glottic exposure in the video laryngoscope group was 100%, which was higher than that in the direct laryngoscope group (63.5%). Even the percentage of patients with Wilson-Cormack-Lehane classification I-II by the junior anesthetist in the video laryngoscope group (100%) was also higher than that by the senior anesthetist in the direct laryngoscope group (64.4%), indicating that the video laryngoscope could effectively improve glottic exposure in the tracheal intubation of patients undergoing elective surgery. The above results were similar to those reported in the literature addressing video laryngoscope use for difficult airways and video laryngoscope use by novice operators [9–11]. The results demonstrated that the visual equipment also had the advantage of providing good glottic exposure in patients undergoing elective surgery.

Similar to the success rate of 93.6% for one-time intubation with video laryngoscope reported in a study by Ibinson et al. [12], our results demonstrated that the intubation success rate for one-time intubation in the video laryngoscope group (96.1%) was higher than that in the direct laryngoscope group with the senior anesthetist (90.1%). The total intubation success rate in the two trials of the video laryngoscope group was 100%, which was higher than that of the direct laryngoscope group (94.5%). When using the direct laryngoscope, after intubation was blocked, the intubation operator had to switch to a video laryngoscope to complete tracheal intubation in 7 cases. However, re-intubation could have been avoided if the video laryngoscope was used in the first attempt. Earlier studies reported that, despite the significant advantage of glottic exposure using the video laryngoscope, the limitation was that, even if the operator clearly observed the glottis, difficulties may occur when placing the endotracheal tubes [5]; however, appropriate limits on glottic exposure grading when using a direct laryngoscope may be better for tracheal intubation [13]. The reason was that the opening of the oral cavity was reduced during intubation using the video laryngoscope and the operable space of the oropharynx was also narrowed while the angle adjustment of the catheter in the oropharyngeal cavity became more difficult. Some investigators have used special equipment, such as a fibrobronchoscope or Infrared Red Intubation System (IRRIS) equipment, to assist in video laryngoscope intubation, which achieved more ideal outcomes [14, 15]. However, the use of such equipment would result in an increase in complexity and cost of conventional intubation, which is not conducive to regular large-scale application. Systemic evaluations performed by Lewis et al. recognized the value of visual intubation devices in improving the success rate of intubation of difficult airways. However, they did not thoroughly analyze the differences in intubation success rates and the differences in postoperative complications of the different intubation devices for tracheal intubation in patients with non-difficult airways [16]. To explore reasons for this, a large number of studies have instead focused on the following: 1) the comparison of different types of laryngoscopes in the treatment of airway conditions in selected settings such as in an emergency, in obese patients, during cardiopulmonary resuscitation, during double-lumen intubation, among others [17–20]; 2) the salvage value of the video laryngoscope after the first intubation failure [21]; or 3) the difference in dealing with difficult airways between selected special methods and the video laryngoscope [22].

In our study, 6 cases of oropharyngeal hemorrhage, 2 cases of lip injury, and 1 case of incisor injury occurred after intubation in the direct laryngoscope group. However, only 2 cases of lip injury occurred in the video laryngoscope group. With respect to complications on postoperative day 1, obvious sound change could be heard in 14 patients in the direct laryngoscope group but could be heard in only 5 patients (2.8%) in the video laryngoscope group. From an anatomical perspective, with the head in a natural position, the respiratory tract forms four axes that form angles with one another. When the head is tilted back, the pharynx axis, the laryngeal axis, and the tracheal axis become aligned, which is advantageous for opening the glottis directly. However, the increased tissue tension of the throat caused by lifting the mandible using the direct laryngoscope leads to throat damage by the intubation device and catheter. Many previous studies have demonstrated that the video laryngoscope exerts a lower lifting force on the mandible than a direct laryngoscope in both normal and difficult airways [23–25]. Thus, when the endotracheal tube is inserted effectively, it decreases tension in throat tissue and reduces the damage caused by tracheal intubation. Because the sample size calculation criteria in this study were based on the success rate of tracheal intubation as a standard, there was no significant difference in the incidence of serious complications. However, we believe that if the program expanded the sample size, it is highly likely that differences would emerge in the incidence of serious complications.

All 360 cases were included in the regression analysis, and glottic exposure classification and catheter shape angle were independent risk factors for ≥2 failures. Receiver operating characteristic curve analysis was performed on the measured values of the shape angle, with intubation failure as the state variable. The stratified analysis was performed using the largest Youden index corresponding to the shape angle (86.5°) as the critical point, of which the difference was statistically significant, prompting further binary logistic regression analysis. However, the type of laryngoscope itself was not a decisive factor in intubation failure. We believe that, regardless of whether a video laryngoscope, direct laryngoscope, or another type of visual intubation device is used, the success rate of intubation of non-difficult patients undergoing elective surgery can be increased as long as the level of glottic exposure can be improved. An excessively curved catheter may result in its inability to be adjusted within the mouth to face the glottis. In this case, the catheter must be removed for reshaping.

Similarly, laryngoscope types were not risk factors for postoperative complications and complications that lasted for ≥2 days. However, prolonged time of glottic exposure was an independent risk factor for postoperative complications and complications lasting > 2 days. In operations requiring tracheal intubation, the operator adjusts the position of the laryngoscope in the event of difficulty when the glottis is exposed. Similar to previous conclusions, we found that the longer the process, the greater the risk that the laryngoscope lens can damage the throat tissue. Therefore, any intubation device that can effectively shorten glottic exposure time can effectively mitigate the postoperative complications of intubation.

At the time of design, we randomly assigned the anesthetists’ seniority, hoping to analyze whether anesthetists with more extensive clinical experience demonstrated better intubation ability than junior anesthetists. Unexpectedly, however, there was no significant difference in the use of special equipment such as video laryngoscope, by senior or junior anesthetists to perform intubation of non-difficult patients. The one-time intubation success rates of the junior anesthetists were higher than that of senior anesthetists (97.7% versus 94.5%). This result revealed that the method of operation of the video laryngoscope was very different from that of traditional tracheal intubation. Without receiving extensive training, however, most physicians may still use methods for the direct laryngoscope when initially operating a video laryngoscope. With the availability of such equipment, senior anesthetists and junior anesthetists were essentially at the same starting point. Ambrosio et al. studied first-year resident physicians using the video laryngoscope to approach difficult airways. They found that after learning methods involving both the video laryngoscope and direct laryngoscope, the anesthetists were significantly better at using the video laryngoscope than the direct laryngoscope [26]. Moreover, there were similar conclusions in the comparison of the two types of laryngoscopes among intern anesthetists [27]. However, the results of the present study may have been affected by the volume of intubations performed, in that the junior anesthetists had more opportunities for clinical intubations in the country where the study was performed. Although their experiences were still not as extensive as the senior anesthetists, the junior anesthetists experienced a high frequency of clinical intubations. Therefore, this result may not apply in other countries or regions with low frequencies. However, it is worth noting that there is a direct correlation between the proficiency of an intubation device and its effect in use [28]. In a national survey conducted in the United Kingdom, 91% of anesthesiology departments and 50% of intensive care units were equipped with video laryngoscopes in public hospitals; nevertheless, they were not prevalent in emergency or pediatric departments, or private hospitals [29]. The promotion of visual intubation equipment should not be limited to operating rooms.

Our study had limitations. First, a regression equation was not calculated for the risk factors for video laryngoscope intubation to improve the efficiency of video laryngoscope intubation. Because the sample involved in this study were regional cases, anatomical data may not be applicable due to differences in ethnicity. Second, only abdominal surgeries were included in the study, which did not involve surgeries that may affect the airway, such as neck surgery and neurosurgery. Therefore, the results of this investigation may still require many follow-up studies to continue the validation analysis.

## Conclusions

Intubation using the video laryngoscope significantly improved the success rate of intubation and significantly lowered postoperative complications associated with intubation compared with direct laryngoscope in patients with non-difficult airways. The use of video laryngoscope is worth considering for intubation of non-difficult airways due to its ease of use and satisfactory safety profile.

## Abbreviations

ASA: American Society of Anesthesiologists
IRRIS: Infrared Red Intubation System
PEtCO_2_: End-tidal carbon dioxide

## References

[1] Tabari M, Soltani G, Zirak N, Alipour M, Khazaeni K (2013). Comparison of effectiveness of betamethasone gel applied to the tracheal tube and IV dexamethasone on postoperative sore throat: a randomized controlled trial. Iran J Otorhinolaryngol.

[2] Cooper RM, Pacey JA, Bishop MJ, McCluskey SA (2005). Early clinical experience with a new videolaryngoscope (GlideScope) in 728 patients. Can J Anaesth.

[3] Hirabayashi Y, Hakozaki T, Fujisawa K, Yamada M, Suzuki H, Satoh M (2007). Masui..

[4] Apfelbaum JL, Silverstein JH, Chung FF, Connis RT, Fillmore RB, Hunt SE (2013). Practice guidelines for postanesthetic care: an updated report by the American Society of Anesthesiologists Task Force on Postanesthetic care. Anesthesiology..

[5] Serocki G, Bein B, Scholz J, Dorges V (2010). Management of the predicted difficult airway: a comparison of conventional blade laryngoscopy with video-assisted blade laryngoscopy and the GlideScope. Eur J Anaesthesiol.

[6] Jungbauer A, Schumann M, Brunkhorst V, Borgers A, Groeben H (2009). Expected difficult tracheal intubation: a prospective comparison of direct laryngoscopy and video laryngoscopy in 200 patients. Br J Anaesth.

[7] Dong J, Gao L, Lu W, Xu Z, Zheng J (2014). Pharmacological interventions for acceleration of the onset time of rocuronium: a meta-analysis. PLoS One.

[8] Lee S, Ro YJ, Koh WU, Nishiyama T, Yang HS (2016). The neuromuscular effects of rocuronium under sevoflurane-remifentanil or propofol-remifentanil anesthesia: a randomized clinical comparative study in an Asian population. BMC Anesthesiol.

[9] Mahran EA, Hassan ME (2016). Comparative randomised study of GlideScope((R)) video laryngoscope versus flexible fibre-optic bronchoscope for awake nasal intubation of oropharyngeal cancer patients with anticipated difficult intubation. Indian J Anaesth.

[10] Abdellatif AA, Ali MA (2014). GlideScope videolaryngoscope versus flexible fiberoptic bronchoscope for awake intubation of morbidly obese patient with predicted difficult intubation. Middle East J Anaesthesiol.

[11] Wang PK, Huang CC, Lee Y, Chen TY, Lai HY (2013). Comparison of 3 video laryngoscopes with the Macintosh in a manikin with easy and difficult simulated airways. Am J Emerg Med.

[12] Ibinson JW, Ezaru CS, Cormican DS, Mangione MP (2014). GlideScope use improves intubation success rates: an observational study using propensity score matching. BMC Anesthesiol.

[13] Gu Y, Robert J, Kovacs G, Milne AD, Morris I, Hung O (2016). A deliberately restricted laryngeal view with the GlideScope(R) video laryngoscope is associated with faster and easier tracheal intubation when compared with a full glottic view: a randomized clinical trial. Can J Anaesth.

[14] Liu WF, He HP, Xie WX, Weng PQ, Li SY (2012). Effects of different nasotracheal intubations in obstructive sleep apnea hypopnea syndrome patients with uvulopalatopharyngoplasty. Zhonghua Yi Xue Za Zhi.

[15] Biro P, Fried E, Schlaepfer M, Kristensen MS (2018). A new retrograde transillumination technique for videolaryngoscopic tracheal intubation. Anaesthesia..

[16] Lewis SR, Butler AR, Parker J, Cook TM, Smith AF (2016). Videolaryngoscopy versus direct laryngoscopy for adult patients requiring tracheal intubation. Cochrane Database Syst Rev.

[17] Sakles JC, Douglas MJK, Hypes CD, Patanwala AE, Mosier JM (2017). Management of Patients with predicted difficult Airways in an Academic Emergency Department. J Emerg Med.

[18] Yumul R, Elvir-Lazo OL, White PF, Sloninsky A, Kaplan M, Kariger R (2016). Comparison of three video laryngoscopy devices to direct laryngoscopy for intubating obese patients: a randomized controlled trial. J Clin Anesth.

[19] Kim JW, Park SO, Lee KR, Hong DY, Baek KJ, Lee YH (2016). Video laryngoscopy vs. direct laryngoscopy: which should be chosen for endotracheal intubation during cardiopulmonary resuscitation? A prospective randomized controlled study of experienced intubators. Resuscitation..

[20] Belze O, Lepage E, Bazin Y, Kerourin P, Fusciardi J, Remerand F (2017). Glidescope versus Airtraq DL for double-lumen tracheal tube insertion in patients with a predicted or known difficult airway: a randomised study. Eur J Anaesthesiol.

[21] Aziz MF, Brambrink AM, Healy DW, Willett AW, Shanks A, Tremper T (2016). Success of intubation rescue techniques after failed direct laryngoscopy in adults: a retrospective comparative analysis from the multicenter perioperative outcomes group. Anesthesiology..

[22] Aziz MF, Abrons RO, Cattano D, Bayman EO, Swanson DE, Hagberg CA (2016). First-attempt intubation success of video laryngoscopy in patients with anticipated difficult direct laryngoscopy: a multicenter randomized controlled trial comparing the C-MAC D-blade versus the GlideScope in a mixed provider and diverse patient population. Anesth Analg.

[23] Carassiti M, Biselli V, Cecchini S, Zanzonico R, Schena E, Silvestri S (2013). Force and pressure distribution using Macintosh and GlideScope laryngoscopes in normal airway: an in vivo study. Minerva Anestesiol.

[24] Fiadjoe JE, Stricker P (2012). Force and pressure distribution using Macintosh and GlideScope laryngoscopes. Br J Anaesth.

[25] Carassiti M, Zanzonico R, Cecchini S, Silvestri S, Cataldo R, Agro FE (2012). Force and pressure distribution using Macintosh and GlideScope laryngoscopes in normal and difficult airways: a manikin study. Br J Anaesth.

[26] Ambrosio A, Pfannenstiel T, Bach K, Cornelissen C, Gaconnet C, Brigger MT (2014). Difficult airway management for novice physicians: a randomized trial comparing direct and video-assisted laryngoscopy. Otolaryngol Head Neck Surg.

[27] Aqil M, Khan MU, Hussain A, Khokhar RS, Mansoor S, Alzahrani T (2016). Routine use of Glidescope and Macintosh laryngoscope by trainee anesthetists. J Coll Physicians Surg Pak.

[28] Russell KA, Brook CD, Platt MP, Grillone GA, Aliphas A, Noordzij JP (2017). The benefits and limitations of targeted training in flexible Transnasal laryngoscopy diagnosis. JAMA Otolaryngol Head Neck Surg.

[29] Cook TM, Kelly FE (2017). A national survey of videolaryngoscopy in the United Kingdom. Br J Anaesth.

